# Applying a Weight-of-Evidence Approach to Evaluate Relevance of Molecular Landscapes in the Exposure-Disease Paradigm

**DOI:** 10.1155/2015/515798

**Published:** 2015-08-03

**Authors:** Sherilyn A. Gross, Kristen M. Fedak

**Affiliations:** ^1^Cardno ChemRisk, 4840 Pearl East Circle 300 W., Boulder, CO 80304, USA; ^2^Department of Environmental & Radiological Health Sciences, Colorado State University, Fort Collins, CO 80523, USA

## Abstract

Information on polymorphisms, mutations, and epigenetic events has become increasingly important in our understanding of molecular mechanisms associated with exposures-disease outcomes. Molecular landscapes can be developed to illustrate the molecular characteristics for environmental carcinogens as well as associated disease outcomes, although comparison of these molecular landscapes can often be difficult to navigate. We developed a method to organize these molecular data that uses a weight-of-evidence approach to rank overlapping molecular events by relative importance for susceptibility to an exposure-disease paradigm. To illustrate the usefulness of this approach, we discuss the example of benzene as an environmental carcinogen and myelodysplastic syndrome (MDS) as a causative disease endpoint. Using this weight-of-evidence method, we found overlapping polymorphisms in the genes for the metabolic enzymes *GST* and *NQO1*, both of which may infer risk of benzene-induced MDS. Polymorphisms in the tumor suppressor gene, *TP53*, and the inflammatory cytokine gene, *TNF-α*, were also noted, albeit inferring opposing outcomes. The alleles identified in the DNA repair gene *RAD51* indicated an increased risk for MDS in MDS patients and low blood cell counts in benzene-exposed workers. We propose the weight-of-evidence approach as a tool to assist in organizing the sea of emerging molecular data in exposure-disease paradigms.

## 1. Introduction

In an idealistic view of carcinogenesis, the molecular initiation of a disease process can be directly tied to a genetic mutation or chromosome event caused by environmental exposure to a carcinogen. However, a single event is usually insufficient to induce cancer and other secondary events such as additional gene mutations and/or chromosome changes are usually required [1]. These additional events occur during the latency period of the disease and the progression of the disease is a function of individual susceptibility and gene-environment interactions [2].

Further, in an idealistic view of a cause and effect relationship between an environmental carcinogen and disease endpoints, exposure to the environmental carcinogen would remain evident at the time that the disease manifests. For example, a causal link has been established between environmental exposure to aflatoxin, DNA modification, and the manifestation of liver cancer [3]. Unfortunately, the aflatoxin biomarker of exposure-evidence of disease paradigm is unique and does not represent the typical template for most exposure-disease paradigms.

Herein, we asked the following: if exposure to a particular environmental carcinogen is implicated in the cause of a disease but the exposure is not evident at the time of disease diagnosis, what additional molecular events (e.g., genetic polymorphisms, genetic mutations, and/or epigenetic events) can be linked to the exposure-disease paradigm? Further, what additional mutational events can be linked to disease progression, as not every and not all exposures lead to disease outcomes? We propose that a “weight-of-evidence” (WoE) approach can be applied to compile evidence from multiple sources in the published literature to create a molecular landscape for the environmental carcinogen and for the disease endpoint in question. Herein, we describe the proposed WoE approach for navigating the molecular landscapes of the exposure-disease paradigm. We discuss this application in the context of benzene-induced myelodysplastic syndrome (MDS), to demonstrate how this approach can be used to identify and assign relevance to molecular events associated with both exposure and outcome, taking the multistep process of carcinogenesis into consideration. Findings from this approach may be useful in identifying a biomarker for a specific environmental chemical, identifying a molecular endpoint to be used in future molecular epidemiology studies, providing support for traditional epidemiology in establishing causal inference, and/or identifying molecular events that are important for those individuals susceptible to a specific exposure-disease paradigm.

## 2. Background

While there are multiple types of mutations identified in cancers, it has been suggested that frank carcinogenesis occurs only when cells acquire defects in the following six key areas of cellular control [2]:

1. sustainable cellular growth independent of a growth signal,
2. abnormality in proteins that regulate the cell cycle,
3. loss of the ability to respond to programmed cell death,
4. cellular immortalization marked by the retention of telomeres,
5. continuous blood supply through sustained angiogenesis,
6. loss of adhesion ability resulting in tissue invasion and metastasis.

The interval between the exposure to a carcinogen and the manifestation of disease allows time for these molecular changes to occur.* Latency *is technically defined as the period of apparent inactivity between the time of first exposure to a causative agent and the time for response, or the first clinical manifestation of the disease [4, 5]. The outwardly observable effects of many carcinogens in humans are typically not seen until after 15- or 20-year latency periods [5, 6]. Latency periods have also been described as the “time interval between disease occurrence and detection of disease either by medical testing or by emergence of symptoms,” a definition that suggests that latency can be shortened with improvement on detection methods [6]. Thus, cancer is a disease of latency and in many cases a natural artifact of evolution without any known cause. Molecular landscapes have been used to help establish the six key defects that are the hallmark of frank carcinogenesis; however, understanding the timing and order of occurrence of these molecular events during the latency period is ongoing.

WoE refers to the interpretive methods commonly applied to bodies of literature when conducting hazard and risk assessments. These types of approaches have been used for decades by academics, practitioners, and regulatory agencies in both formal and informal risk assessment processes to establish understandings of causality, discuss chemical hazards, and set regulatory action levels for exposure or contamination. For example, the International Agency for Research on Cancer (IARC) applies a WoE approach to evaluating carcinogenic risk, which they describe in the preamble to their* Monographs on the Evaluation of Carcinogenic Risk to Humans* [7, 8]. Similarly, the U.S. Environmental Protection Agency's (EPA's) Integrated Risk Information System (IRIS) program utilizes WoE approaches within their toxicological profiles and health risk assessments, which categorize carcinogenicity potential using a 5-point WoE scale that was established in 1986 [9, 10].

Yet, while WoE is not a new concept, scientists have faced challenges in the fact that the meaning of “weight-of-evidence” in its practical application is not necessarily clear or well defined [11]. Recently, researchers have pushed the methodological discussions of WoE one step further into a quality evaluation, thereby pulling theoretical frameworks into practical application [12]. For example, the Office of Health Assessment and Translation (OHAT) within NIEHS recently integrated traditional WoE concepts with systematic review guidelines to develop a complex framework for conducting literature-based health assessments [13]. The OHAT approach provides transparent, nonsubjective guidelines and methodology for comparing and contrasting data that comes from a wide range of study design types (e.g.,* in vitro* and* in vivo* toxicology, various epidemiological designs, and molecular studies), considering broader aspects of quantitative study quality [13]. WoE approaches have also been used to provide direction for research protocols when causation has already been established. For example, Zelenka et al. [14] proposed a WoE framework for selecting the most appropriate biomarkers of exposure to use for biomonitoring analysis. The authors presented an example wherein they used the framework to evaluate six biomarkers for benzene exposures over 1 ppm over an 8-hour time frame in occupational settings.

Herein, we suggest another application of WoE, which combines traditional methods for establishing causal inference with more recent uses in providing direction for molecular research. While no interpretive WoE method can eliminate the need for some level of expert judgment, WoE frameworks such as the one we suggest herein can help reduce subjectivity and increase transparency in the vast body of literature, which can lead to greater understanding of a particular exposure-disease paradigm than might be possible without the aid of such a tool.

Benzene is a known environmental carcinogen given adequate dose and duration of exposure [15]. Crude oil normally contains a varying composition of petroleum hydrocarbons, including benzene [16]. As such, benzene remains an unavoidable component of gasoline products and is a major product in the petroleum refining industry [17, 18]. The recent increase in unconventional exploration and production of oil and gas near residential communities across the United States may lead to increased opportunities for benzene contamination of valued air and water resources [19].

Benzene is also a known leukemogen but the molecular events required for the development of benzene-induced leukemia occur over an 8- to 15-year latency period [20, 21]. As part of this latency period, however, investigators have described a benzene-induced “preleukemic state” occurring in benzene-exposed individuals prior to diagnosis of a leukemic state [22]. We now know that the preleukemia described in benzene-exposed individuals decades earlier is now considered as MDS [23]. In turn, the leukemias have been classified as myeloid and lymphoid with numerous heterogeneous groups of leukemia subtypes within each classification, all of which have distinct molecular landscapes, clinical features, etiologies, prognoses, and therapy [23].

Recent investigation has revealed that MDS is the most sensitive dose-dependent carcinogenic endpoint following benzene exposure in the occupational setting, with MDS occurring in some petroleum workers at peak exposure levels as low as 3 parts per million (ppm) [22]. MDS represents a small set of heterogeneous clonal diseases of the bone marrow that have been associated with a variety of exposures beyond benzene, including solvents, agricultural chemicals, cigarette smoke, chemotherapy, and ionizing radiation, but MDS can also occur spontaneously [23].

Regarding markers for benzene-induced disease, several biomarkers of exposure to benzene exist, including urinary metabolites of benzene* s*-phenylmercapturic acid (sPMA), benzene-induced depression of peripheral blood parameters (e.g., neutrophils and MPV), and adducts to hemoglobin and albumin [24–28]. While these biomarkers are useful for monitoring exposure in occupational cohorts or other types of suspected-exposure scenarios, the biomarkers are transient and usually disappear sometime shortly after cessation of exposure [14, 24]. Therefore, benzene represents an environmental carcinogen in which the molecular mechanisms associated with disease progression are not clearly defined or easily elucidated using these transient biomarkers of exposure.

In an effort to examine molecular events associated with MDS disease progression, large scale genome-wide associated studies have been conducted with hundreds of MDS patients [29–31]. In turn, genome-wide associated studies have also been conducted in benzene-exposed workers [32, 33]. Findings from these studies and other published literature can provide an opportunity to compare the respective molecular landscapes (e.g., genetic polymorphisms, somatic genetic mutations, and epigenetic changes).

In considering the molecular landscapes, it is important to differentiate a clear definition for each molecular piece of the landscape. Polymorphisms are inherent to the genome and were originally used to describe variations in shape and form that distinguish normal proteins within a species from each other which makes each human genome unique. Because polymorphism can cause extreme variation in protein function, some polymorphisms can infer genetic susceptibility to a certain agent or disease outcomes whereas other polymorphisms can infer resistance. A mutation, conversely, is a permanent change of the DNA sequence following an exposure or event. Mutations result from unrepaired damage to DNA that occurs due to errors in the process of replication or from the insertion or deletion of segments of DNA by exogenous agents. Finally, epigenetic events are those that cause modification of the genome without modification of the DNA itself. Epigenetic events can result in a change in the expression of the underlying genetic trait by altering the timing and quantity of expression at key points in time (i.e., variation in timing of the expression of a functional protein).

## 3. Methods

**Figure 1:**
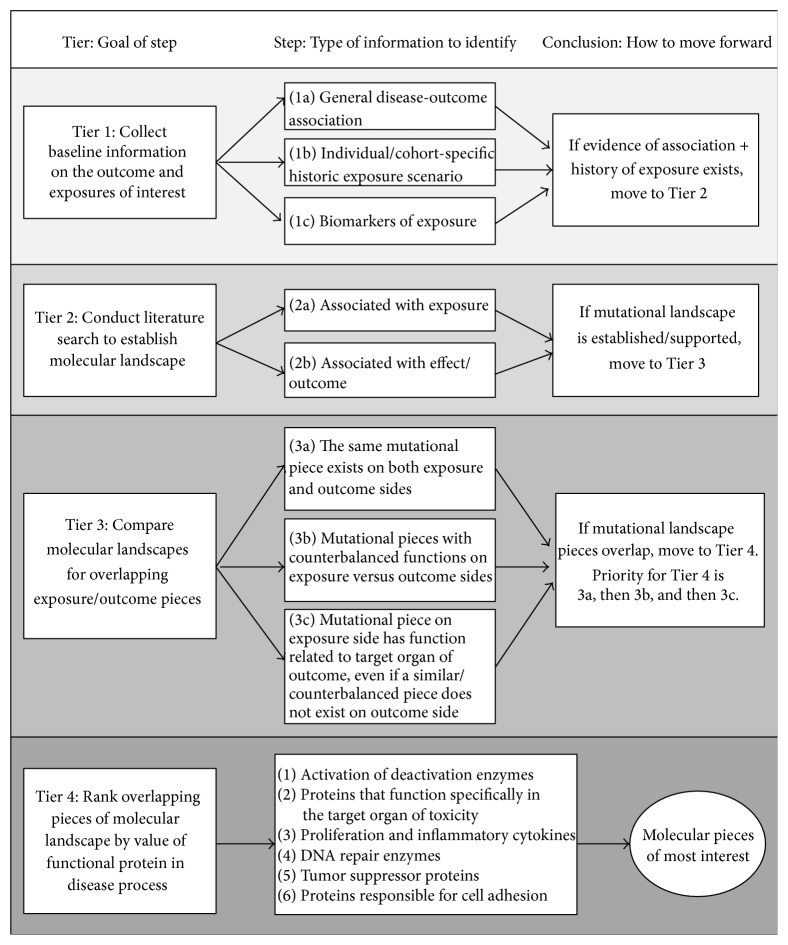
Weight-of-evidence approach for navigating the molecular landscape. This figure illustrates the stepwise approach used to sort and weigh evidence related to the molecular landscape of an exposure-disease paradigm, starting with the top, left-hand block and moving sequentially to the bottom, right-hand block.

We developed a WoE approach as a tool to navigate the molecular landscape of any given exposure-disease paradigm. The approach is demonstrated in Figure 1.

The first tier of the WoE approach involves collecting baseline information on the outcome and exposures of interest. The goal of this tier is to determine if enough baseline evidence exists to illustrate that a relationship may exist between an exposure and outcome to warrant more detailed, intensive molecular investigation. Without documented indications that the outcome of interest may be associated with the suspected environmental carcinogen, and that exposure to that agent likely occurred or is occurring in the study population, molecular investigations related to those exposures and outcomes are not a useful tool. The first step within this tier is to perform a literature search to determine whether the disease endpoint in question has been associated with the environmental carcinogen of interest in published, peer-reviewed literature. This can include both epidemiological and toxicological studies, though one should generally be cautious of study quality and consider the overall strength of the body of literature on this association. Simultaneously, one should determine whether the individual or group of individuals in question had a historic exposure or potential/likely exposure to a suspected toxic agent prior to the development of the disease endpoint. At the most simplistic level, this can be achieved through establishing work history summaries or definitions of cohort groups based on job categories (using oral recall, social security records, or jobsite employment records). More detailed, specific methods to establish an exposure history should also be employed if data is available, such as exposure monitoring or industrial hygiene records. Additionally, if exposure to the environmental carcinogen was documented at the time of alleged exposure, records indicating biological evidence of exposure (e.g., evidence of metabolism in bodily fluids) add an additional level of certainty. An understanding of available biomarkers of exposure to the exposure agent and the residence time of those markers following exposure cessation is also useful at this time.

Once there is an understanding of a reasonably assumed exposure that occurred in the study population as well as evidence of an association between the exposure agent and the outcome, one can confidently move into the second tier of the WoE approach. This tier involves conducting a literature search to establish the molecular landscape associated with the exposure-outcome paradigm of interest. A comprehensive, systematic literature search should be conducted to identify peer-reviewed published literature related to molecular events (e.g., genetic polymorphisms, genetic mutations, and epigenetic changes) for both the environmental carcinogen and the disease endpoint. Identified information on the molecular event and the function or effect of that event can be organized into lists or tables, such as those shown in Tables 1(a) and 1(b). If no information is identified related to either the exposure or the effect/outcome side of the molecular landscape, one cannot move any further through the WoE framework, as this indicates that more research must be conducted related to identifying and characterizing the molecular landscapes.

The goal of the third tier is to compare the molecular landscapes for both the environmental carcinogen and the disease endpoint identified during the second tier. To do this, the pieces of the molecular landscapes should be compared side by side to identifying overlapping molecular events. These overlaps can occur in a number of ways. For example, is there a single nucleotide polymorphism (SNP) on the exposure side that represents the same change in protein function as the SNP from the outcome side (e.g., DNA codes for the same amino acid sequence used to build the protein)? Or, on the other hand, are there SNPs that are counterbalanced across the exposure and outcome sides? Direct overlaps of the same molecular events on both the exposure and outcome side represent the highest priority for further investigation in the fourth tier. Counterbalanced or complementary overlaps that result in counterbalancing functions represent a second priority level for the fourth tier. Finally, there may be mutational pieces seen on the exposure side that result in a functional change related to the target organ in which the outcome is seen. Even if a similar or counterbalanced/complementary piece is not seen on the outcome side, these molecular pieces could be of interest and should be carried into the fourth tier of the WoE approach. If comparison of the molecular landscape at the tier 3 level reveals no overlaps, one must consider whether this implies that the molecular landscape has not yet been fully developed, in which case more researches into the molecular changes associated with the agent of exposure and/or the disease endpoint are warranted, or that the exposure-disease association of interest is not supported by the molecular landscapes.

Finally, once the molecular landscapes have been narrowed down to only the overlapping pieces, the fourth tier of the WoE approach can be implemented. This tier involves ranking overlapping pieces of the molecular landscape by value of the functional protein affected by that molecular event in the disease process. When investigating exposure and effects, the priority for the functionalities is determined based on the importance of that change to susceptibility to carcinogenesis/disease progresses and the specificity that the change implies for the specific exposure-disease paradigm versus a generic change that is seen in all cancers.

Base on our professional judgment, we suggest an initial priority level for polymorphisms and mutations as follows (from greatest to least relevant):

1. activation and deactivation enzymes of environmental carcinogens,
2. proteins that function specifically in the target organ of toxicity,
3. proliferation and inflammatory cytokines,
4. DNA repair enzymes,
5. tumor suppressor proteins,
6. proteins responsible for cell adhesion.

Similarly, we suggest an initial priority level epigenetic event as follows (from greatest to least relevant):

1. hypo- or hypermethylation (modify timing of DNA expression into protein),
2. hypo- or hyperacetylation (modify DNA expression into protein),
3. histone modification (open DNA reading frame).

The result of the fourth tier, as well as the overall result of the WoE approach, is a list of related and relevant molecular events associated with the exposure and the outcome side of a given exposure-disease paradigm that can be used to indicate a more likely association has occurred between that exposure and the outcome. These molecular events warrant the highest level of further consideration within the sea of information related to the given relationship.

We suggest that common polymorphisms, genetic mutations, and epigenetic events be given the highest WoE ranking if the environmental carcinogen in question is directly or indirectly toxic through DNA adduct formation (e.g., through reactive oxygen), and then DNA repair mechanisms should be elevated, as genetic changes in DNA repair genes would be expected in the process of carcinogenesis in any tissue [2]. We anticipate that epigenetic events will become more important within the next few years as researchers develop new methods of analysis to correlate changes in methylation state and alterations in the timing of expression of a functional protein (e.g., phenotype). We trust that gaps in our current understanding of this process such as how epigenetic events relate to downstream proteins involved in the exposure-disease paradigm will be revealed [31].

## 4. Results

To illustrate how this WoE approach works, we applied the methods to the exposure-disease paradigm of benzene and MDS. In this specific scenario, we chose to rank the overlapping molecular landscape on the basis of susceptibility to benzene-induced MDS. We started our WoE approach at the second tier, because the first tier step of determining whether evidence exists for an assumed association between benzene and MDS has been done by other researchers, and this was a hypothetical exercise so there was no individual or cohort for which exposure assumptions needed to be established [33]. A literature search was conducted using PubMed, to identify published findings that discussed the molecular landscape associated with environmental exposure to benzene and molecular mechanisms associated with benzene-induced toxicity. An independent literature search was performed to identify the molecular landscape associated with the disease progression to MDS. The PubMed searches were conducted for illustration purposes and were not meant to be a comprehensive search but rather to provide enough collective information to be useful for demonstrating how the WoE approach we developed can be applied to help bring order and relevance to molecular information.

The known molecular landscapes for MDS and benzene, respectively, as determined by our literature search are displayed in Tables 1(a) and 1(b).

Where possible, we used the published literature to determine if the molecular events identified for benzene and MDS represented the same type of change in protein function (e.g., enhanced function, inhibition of function, or no change in function). Molecular landscapes (e.g., polymorphisms, mutations, epigenetic events) for MDS and benzene ranked by the WoE approach are shown in Table 2.

### 4.1. Common Polymorphisms

By overlapping the molecular landscapes for MDS and benzene, we identified common polymorphisms and ranked them by molecular event of interest (MEoI) based on the criteria outlined in Methods. Our finding showed overlap in the genes for the metabolic enzymes glutathione-*S*-transferase (GST) (GSTT1, GSTM1, and GSTP1) and NAD(P)H:quinone oxidoreductase 1 (NQO1). We also found additional polymorphisms in benzene-exposed workers in cytochrome P4502E1 (CYP2E1) and myeloperoxidase (MPO) but not in MDS patients. These metabolic enzymes have been examined in assays for variations in activity associated with benzene toxicity [34]. For example, the metabolic enzyme CYP2E1 in the liver is responsible for transformation of benzene into its major metabolites hydroquinone (HQ) and catechol (CAT). MPO in bone marrow progenitor cells has been demonstrated to further metabolize HQ to the bone marrow toxin,* para*-benzoquinone (pBQ). Detoxification of benzene metabolites in the liver is controlled by GST genotypes whereas detoxification of pBQ in the bone marrow is thought to occur through the NQO1 enzyme [34]. We found that the MEoI in metabolic enzymes associated with MDS was the* NQO1* germline polymorphism C609T, which results in a lowering of NQO1 enzyme activity, which in turn may result in an increase in susceptibility to MDS in benzene-exposed individuals. However, a NQO1C609T polymorphism has been shown to have no effect in MDS [35]. In addition,* GSTT1* and* GSTM1* are genetic polymorphisms of GST in humans, and a homozygous deletion in these enzymes leads to a complete absence of enzyme activity [36]. It was noted in one report that the GSTM1 genotype may contribute towards progression of MDS [36]. In an evaluation of various polymorphisms in metabolic enzymes in Chinese workers occupationally exposed to benzene, NQO1C609T, GSTT1, and GSTM1 inferred an increased risk of benzene poisoning [37]. Taken together, these findings suggest that GST variant GSTM1 increases the risk for MDS and increase the toxicity of benzene by decreasing the ability to detoxify and eliminate the active metabolite. Although the polymorphism NQO1C609T showed no increased risk of MDS, NQO1 functions in the target organ of toxicity for benzene, the bone marrow [34]. Therefore,* GSTM1* and* NQO1* polymorphisms rank as *R* = 1 and *R* = 2, respectively, on the WoE scale.

Mutations in the tumor suppressor gene* TP53* have been shown to be frequent mutations in human cancers [29]. Several polymorphisms in TP53 have been studied in MDS. For example, it has been shown that the TP53Arg72Pro polymorphism did not differ between MDS and healthy controls, and this particular polymorphism was not associated with clinical and laboratory parameters, disease progression, or overall survival of MDS patients [29]. This suggests that TP53 polymorphism is not involved in increased risk for MDS. However, in one report on benzene-exposed workers, the rs1042522 TP53 polymorphism was associated with decreased granulocytes, decreased CD4 T cells, and decreased B-cells [32]. Because the TP53 polymorphism in benzene-exposed workers did not correlate directly with toxicity and showed no involvement in increased risk for MDS,* TP53* common genetic polymorphism was ranked as *R* = 4 on the WoE scale.

Tumor Necrosis Factor alpha (TNF-*α*) protein is a major regulatory cytokine that plays a role in many immune-mediated diseases and hematologic malignancies [38]. In MDS, the −308A* TNF-α* genetic polymorphism, which increases the transcription level of this inflammatory cytokine, was associated with MDS patients [38]. Overexpression of −308A TNF-*α* protein may also be responsible for promoting a proinflammatory state in benzene-exposed workers; one study showed that only the −238 TNF-*α* and not the −308A TNF-*α* polymorphism was significantly associated with the development of benzene-induced dysplasia and not with an increased risk of MDS [39]. Although −308A TNF-*α* and −238 TNF-*α* showed the opposite effect in these studies, TNF-*α* does play a significant role in the target organ of toxicity [39]. Therefore, TNF-*α* would also rank as *R* = 2 on the WoE scale.

The RAD51 protein plays an important role in DNA repair, meiosis, chromosome segregation, and chromosome stability, and its dysregulation has been associated with multiple diseases [40]. A meta-analysis that was performed on a total of ten studies with MDS patients and controls indicated that −135G/C RAD51 protein was associated with an increased susceptibility to MDS [41]. Similarly, a study conducted in 250 benzene-exposed workers and controls indicated that the −135G/C* RAD51* allele was associated with white blood cell (WBC) counts lower than 4000 *μ*L [32]. Since the −135G/C RAD51 polymorphism has been linked to an increased susceptibility to MDS and the identical polymorphism was also associated with changes in WBC counts in benzene-exposed workers, 135G/C RAD51 could rank high on the WoE scale. However,* RAD51* polymorphisms have been associated with multiple diseases so this lowers the rank to *R* = 3.

### 4.2. MDS Gene Mutations

The* RUNX* gene, also known as AML1, codes for an important transcription factor, “core binding factor” alpha subunit, which is a transcription factor that regulates commitment to erythroid and granulocytic lineages and initiates the terminal differentiation of the myeloid lineage [31]. AML1 is commonly fused to* RUNTX1T1* (ETO) in a chromosome translocation t(8;21)(q22;q22), which is one of the most frequent karyotypes in AML [42].* RUNX* gene mutations have been identified in some cases of MDS and generally infer unfavorable prognosis [30, 31].

### 4.3. Benzene-Induced Epigenetic Events

Interestingly, when* RUNX* methylation status was examined in a cell line treated with the benzene metabolite HQ, researchers found that HQ induced hypermethylation in* RUNX* as well as hypomethylation in RUNTX1T1 [43]. Taken together, the mutation in* RUNX1* in some MDS patients and the* RUNX1* epigenetic event shown in* in vitro* cell culture treated with benzene metabolites may be important if the RUNX1 finding can be reproduced in workers exposed to benzene, especially since this mutation occurs in the target organ of toxicity. We propose that the* RUNX1* mutation in MDS and epigenetic changes shown in an* in vitro* cell culture treated with benzene metabolites rank as *R* = 5 on the WoE scale.

### 4.4. Common Epigenetic Events

DNA methyltransferases (DNMTs) play a key role in establishing and maintaining methylation. DNA methylation is considered to be the initial step in establishing the inactive chromatin state and is critical for maintaining silence (i.e., no gene expression) in protooncogenes [44]. Reduced DNA methylation has been correlated with shorter survival times and transformation from MDS to AML [31]. Among Chinese workers exposed to a mixture of benzene, toluene, and xylene, those who exhibited a loss of function mutation in DNMT3A also showed a downregulation in all DNMTs and there was a dose-dependent decrease in* DNMT3A* gene expression [45]. The epigenetic events demonstrated in MDS patients and in Chinese workers exposed to benzene are interesting, but since the DNMTs function is nonspecific, the common* DNMT* gene mutation ranks as *R* = 6 on the WoE scale.

### 4.5. Benzene Polymorphisms

Polymorphisms in the metabolic enzyme myeloperoxidase (MPO) within the bone marrow, specifically, the balance between MPO and NQO1 and the benzene metabolites, have been studied. Because both of these polymorphic enzymes occur in the target organ for benzene toxicity, they may have a significant effect on susceptibility to benzene toxicity [43]. Interleukin (IL) cytokines, including IL-10, IL-4, IL-12, and IL-1a, play a pivotal role in growth, maturation, and differentiation of blood cells. Therefore, polymorphisms in these regulators that cause an enhanced production, interfere with receptor binding, or inhibit cell function could have a profound effect on the regulation of hematopoietic system as a whole [46]. Given that similar polymorphisms, mutations, or epigenetic events have not been reported in MDS patients, their role in this specific disease process is questionable. Therefore these polymorphisms rank as *R* = 7 and *R* = 8, respectively.

### 4.6. Benzene Gene Mutations

The glycophorin A (GPA) locus codes for an erythroid lineage specific protein with two allelic forms (M and N). When* GPA* alleles were examined in a small group of benzene-exposed workers and control subjects, it was shown that lifetime cumulative exposure to benzene was associated with the NN variant of GPA but not with the N0 variant. It was suggested that the NN mutation occurred in longer-lived bone marrow cells and that the NN variant resulted from a loss of the GPA M allele, possibly through benzene-induced duplication of the N allele. The N0 variant was presumed to occur through point mutations or deletions [47]. Although these results are interesting for benzene-exposed individuals, the role this mutation plays in MDS remains elusive. Therefore, the benzene-induced gene mutation ranks as *R* = 9.

## 5. Discussion

We developed this WoE process in an effort to understand the commonalities between the molecular landscape of an environmental carcinogen and the molecular landscape of a known or suspected disease outcome. We showed how the WoE approach could be used to identify and assign relevance (e.g., rank) to overlapping genetic information associated with susceptibility in an exposure-disease paradigm. We envision that this approach can be modified to identify and rank the most relevant overlapping epigenetic events in an exposure-disease paradigm.

On an individual level, the approach might have practical application for purposes of prevention such as identifying worker susceptibility in an occupational setting. For example, an individual with the potential for high occupational exposure to a specific chemical with a known association with a specific disease outcome could undergo molecular testing to determine whether they are (a) susceptible to initial toxicity from exposure to a specific chemical, (b) susceptible to initiation of molecular events associated with disease progression, or (c) susceptible to a specific disease regardless of exposure. In this scenario, (a) the worker could be monitored for markers of exposure and toxicity to a specific chemical, (b) the worker could be monitored for the development of an identified marker indicative of early disease, or (c) the worker could be evaluated for the presence of molecular events specific to the manifestation to the disease in question. Armed with this type of knowledge, one could take action to prevent or avoid subsequent exposure to the specific chemical or gain awareness and practice avoidance to all known environmental causes of the particular disease in question.

Further, in situations of outward observable disease, many are tempted to ask the following question: “Is this disease due to my past exposures?” With advanced understanding of the relevance of molecular landscapes and of overlapping molecular events between an environmental carcinogen and a specific disease outcome, it may be possible to answer this question in a more definitive way. Caution must be taken, however, when interpreting these molecular events as we do not suggest that the WoE approach to assigning significance to an overlapping molecular landscape should be used as a surrogate for standard epidemiologic methods for determining causation. Rather, we view this method as evidentiary support for causal inference. Further research is needed to understand how molecular landscapes correlate with individual exposures to carcinogens, molecular mechanisms for disease progression, and disease etiology.

The true usefulness of this approach is to further our understanding of molecular epidemiology on a population level. As the field of molecular epidemiology progresses quickly, biomarkers for exposure, disease progression, and disease outcome are becoming more and more prevalent. Given the increasing amount of molecular information that researchers have access to through the published literature, a system such as the WoE approach illustrated herein is a useful tool for sorting, categorizing, and prioritizing the most meaningful information. Using such a framework, researchers can determine ways to take advantage of best practices in identifying exposure scenarios and defining biomarker(s) relevant to the exposure-disease paradigm.

## Figures and Tables

**(a):** 

Gene	Function	Reference
**Somatic mutations**		
CTCF	Zinc finger protein	[30]
FANCL	DNA cross-link repair in Fanconi anemia	[30]
BRCC3	Cohesin	[30]
MPL	Cohesin	[30]
RAD21	Cohesin complex-sister chromatid separation	[30, 31]
SMC1A	Cohesin complex-sister chromatid separation	[30, 31]
SMC3	Cohesin complex-sister chromatid separation	[30, 31]
STAG2	Cohesin complex-sister chromatid separation	[30, 31]
TET2	DNA hydroxymethylation	[30, 31]
IDH1/2	DNA methylation	[30]
DNMT3A	DNA methylation	[30, 31]
SETBP1	Gain of function	[31]
ASXL1	Histone modification	[30, 31]
EZH2	Histone modification	[30, 31]
LAMB4	Loss of expression in cancer with microsatellite instability	[30]
NF1	Ras pathway	[30]
RIT1	Ras pathway activation	[31]
JAK2	Signal transduction	[30, 31]
N-/K-RAS	Signal transduction	[30, 31]
LUC7L2	Spicing	[30]
SF3B1	Spliceosome	[30, 31]
ZRSR2	Spliceosome	[30, 31]
SRSF2	Spliceosome	[30, 31]
U2AF1	Spliceosome	[30, 31]
ETV6	Transcription factor	[30, 31]
IRF1	Transcription factor	[30]
RUNX1	Transcription factor	[30, 31]
CEBPA	Transcription factor myeloid differentiation	[31]
WT1	Transcription factor myeloid differentiation	[31]
TP53	Transcription factor, tumor suppressor	[30, 31]
BCOR/L1	Transcription repressor	[30, 31]
PHF6	Transcription factor	[30]
ATM	Ataxia telangiectasia mutated gene	[30]

**Polymorphisms**		
ATM	Recognizing and repairing DNA lesions	[42]
JAK3	Variants unrelated to MDS	[42]
KDR	Mediates VEGF's responses to angiogenesis	[42]
STK11	Variants unrelated to MDS	[42]
VEGF/VEGFR	Controversial findings with cancer risk	[42]
RAD51	DNA repair	[48]
XRCC5	DNA repair	[48]
XRCC6	DNA repair	[48]
TGF	MDS disease progression	[49]
TNF-*α*	Increase anemia and thrombocytopenia in MDS	[38]
GSTP1	Increased risk in MDS	[35]
GSTT1	Increased risk MDS	[50]
RAD51	Increased risk MDS	[41]
MDR-1	Multidrug resistant, protective against MDS	[36]
TNF-*α*	No effect in MDS	[49]
NQO1	No effect in MDS	[35]
TP53	Polymorphism not involved in MDS	[51]
BCL2L10	Reduced risk MDS	[42]

**(b):** 

Gene	Function	Reference
**Somatic mutations caused by benzene**		
DNMT1	Decreased mRNA expression	[45]
DNMT3A	Decreased mRNA expression	[45]
DNMT3B	Decreased mRNA expression	[45]
MBD2	Decreased mRNA expression	[45]
PARP1	Decreased mRNA expression	[45]
p15	Hypermethylation	[52]
MAGE-1	Hypomethylation	[52]
Glycophorin A	Induction of gene duplication	[47]
RUNX1	Transcription factor	[43]

**Polymorphism of benzene susceptibility**		
BLM	Modulation of DNA repair	[29, 39]
RAD51	Modulation of DNA repair	[29, 39]
TP53	Modulation of DNA repair	[29, 39]
WDR79	Modulation of DNA repair	[29, 39]
WNR	Modulation of DNA repair	[22]
XRCC1	Modulation of DNA repair	[37]
VCAM1	Altered adhesion	[53]
IL-12	Altered function polymorphism	[43]
MPO	Altered function polymorphism	[43]
NQO1	Altered function polymorphism	[52]
IL-10	Cytokine activity	[32]
IL-12A	Cytokine activity	[32]
IL-1a	Cytokine activity	[32]
IL-4	Cytokine activity	[32]
GSTM1	Detoxification of exogenous compounds	[54]
VEGF	Endothelial cytokine	[53]
TNF-*α*	Inflammatory cytokine	[39]
APEX1	Male restricted DNA repair mechanism	[46]
p14	p53 dependent modulation	[55]
p21	p53 dependent modulation	[55]
MSH2	Repair of mismatched DNA	[56]

**Biomarkers of benzene exposure in blood**		
Urinary sPMA	Increases in urine of exposed individuals	[27]
Hemoglobin adducts	4-month duration in blood	[25]
Albumin adducts	Duration in blood unclear	[26, 28]

**Table 2:** Molecular landscapes (polymorphisms, mutations, epigenetic events) for MDS and benzene ranked by WoE.

Molecular landscapes	
Gene	WoE rank
GSMT1	1
NQO1	2
TNF-*α*	2
RAD51	3
TP53	4
RUNX1	5
DNMTs	6
MPO	7
Interleukins	8
GPA	9
